# The Role of Fibrinolytic System in Health and Disease

**DOI:** 10.3390/ijms23095262

**Published:** 2022-05-09

**Authors:** Hau C. Kwaan

**Affiliations:** Division of Hematology-Oncology, Feinberg School of Medicine, Northwestern University, Chicago, IL 60611, USA; h-kwaan@northwestern.edu

**Keywords:** fibrinolysis, plasmin, plasminogen activator, PAI-1, PAI-2, antiplasmin

## Abstract

The fibrinolytic system is composed of the protease plasmin, its precursor plasminogen and their respective activators, tissue-type plasminogen activator (tPA) and urokinase-type plasminogen activator (uPA), counteracted by their inhibitors, plasminogen activator inhibitor type 1 (PAI-1), plasminogen activator inhibitor type 2 (PAI-2), protein C inhibitor (PCI), thrombin activable fibrinolysis inhibitor (TAFI), protease nexin 1 (PN-1) and neuroserpin. The action of plasmin is counteracted by α2-antiplasmin, α2-macroglobulin, TAFI, and other serine protease inhibitors (antithrombin and α2-antitrypsin) and PN-1 (protease nexin 1). These components are essential regulators of many physiologic processes. They are also involved in the pathogenesis of many disorders. Recent advancements in our understanding of these processes enable the opportunity of drug development in treating many of these disorders.

The fibrinolytic system, also known as the plasminogen–plasmin system, is composed of a proteolytic enzyme plasmin (Pm) with its precursor plasminogen (Pg) (Figure 1) [1,2,3,4,5,6,7]. There are two naturally occurring activators of plasminogen, tissue-type plasminogen activator (tPA) and urokinase-type plasminogen activator (uPA). In addition, the conversion from Pg to Pm can be accomplished by other proteases such as streptokinase, staphylokinase and plasmin. The actions of activators are counteracted by inhibitors, plasminogen activator type 1 (PAI-1), plasminogen activator type 2 (PAI-2), protein C inhibitor (PCI), thrombin activable fibrinolysis inhibitor (TAFI), protease nexin 1 (PN-1) and neuroserpin. Pm, on the other hand, are inhibited by α2-antiplasmin, α2-macroglobulin, TAFI and serine protease inhibitors, including antithrombin and α2-antitrypsin, and by PN-1. In addition, there are receptors for plasminogen [8] and for tPA in the form of annexin II [9,10,11], which is co-localized on the cell surface S-100A10 [12], as well as a receptor for uPA, uPAR [13].

When first discovered, the fibrinolytic system was thought to primarily function as a regulator of fibrin formation and breakdown. Soon it was found that it is involved in many physiological and pathological functions (Table 1 and Table 2). A complete review is beyond the scope of this article, but a few examples are shown below.

Components of the Pg–Pm system are involved in the regulation of menstruation and pregnancy [14], with interactions between the gonadotrophins and tPA, uPA and uPAR. tPA is involved in neuronal growth and learning [15], the regulation of the blood–brain barrier [16,17,18,19], and the regulation of glucose metabolism in the brain [20]. Through multiple pathways, fibrinolytic components can modulate host immunity [21,22,23]. PAI-1 is involved in cell senescence [24] and physiological aging [25]. uPA/uPAR and PAI-1 regulate cell motility and migration and thus are important in wound healing [26,27].

In many types of cancer, there is evidence that there is a correlation between uPA, uPAR and PAI-1 and the aggressiveness and metastatic potential in both tumor cell cultures and tumor tissues [28,29,30,31,32,33]. In carcinoma of the breast, elevated levels of uPA and PAI-1 were found to be associated with a worse prognosis [34]. This association was used in the management of the tumor [35,36]. In carcinoma of the pancreas, the postoperative survival of those with high uPA and PAI-1 was found to be 9 months, while those without these markers was 18 months [37]. A poor response to chemotherapy in small-cell carcinoma of the lung was observed in those with high uPAR [38]. In an athymic mouse model, the transfection of PAI-1 to prostate cancer cells (PC-3) was found to inhibit growth and metastasis [39].

Fibrinolysis is a major component of trauma-induced coagulopathy [40]. As hemorrhage is the major cause of death, excessive fibrinolysis has been observed early after injury and showed a negative predictive value of outcome [41,42,43,44,45,46]. However, the status of fibrinolysis rapidly changes to a hypofibrinolytic phase, often referred to as “fibrinolytic shutdown”. Such a temporal change is part of the body’s response to injury. Persistent low fibrinolytic activity is, however, associated with poor outcomes with multi-organ failure. In one study, patients with low fibrinolytic activity for 7 days had an eightfold higher mortality rate than those whose fibrinolytic activity recovered [47]. In another study, a threefold higher mortality was seen in those with persistent fibrinolytic shutdown at 24 h after injury [48]. Furthermore, the fibrinolytic components play a major role in the pathogenesis of intracranial hemorrhage in trauma patients [40]. Notably, hyperfibrinolysis carries with it a poor prognosis. This is due in part to a breakdown of the blood–brain barrier [16,18,40], as discussed below.

In acute and chronic stress, the hemostatic balance, including endothelial activation, the activation of coagulation and altered fibrinolytic balance, are altered [49]. These changes are pro-thrombotic and hypofibrinolytic with an increase in PAI-1.

Impaired fibrinolysis has been observed in depression with an increase in PAI-1 [50]. Fibrinolysis is an important factor in brain remodeling [51]. Biomarkers for depression are correlated with hypofibrinolysis.

Neurologic functions are another area where tPA and PAI-1 are involved. A wide range of these functions includes ovulation [52], embryogenesis [53], neuronal migration [54], learning [55], the degradation of amyloid [16], stress/fear response [56], and the regulation of the blood–brain barrier [10,12,34]. Notably, tPA increases the permeability of the blood–brain barrier in both a plasmin-dependent and a plasmin-independent pathway. Plasmin activates metalloproteinase directly. Alternatively, tPA can directly activate latent platelet-derived growth factor CC (PDGF-CC), and the tPA–PAI-1 complex activates PDGF receptor alpha (PDGFRa), thus signaling intracellular PI 3K, Ras MAPK, p38 MAPK and PLc-g. These signal an increase in vascular permeability and open the blood–brain barrier [16,18,57,58].

Clinically, these characteristics of tPA are seen as adverse effects. In the treatment of acute ischemic stroke, tPA can increase the risk of hemorrhagic conversion. In nine clinical trials [59,60], hemorrhagic conversion resulted in severe intracranial hemorrhage within 24–36 h in 6.8% of patients, while this figure is 1% in those not receiving tPA, an increase of over fivefold. Fatal intercranial hemorrhage within 7 days occurred in 2.7% of the tPA-treated patients versus 0.4% in those not treated, an over sixfold increase.

In the cardiovascular system, PAI-1 is elevated in cardiovascular diseases [61,62,63] and in metabolic syndrome [64]. In a clinical trial assessing the dietary intake of saturated fatty acids, PAI-1 concentrations were twofold higher in participants at increased risk for cardiometabolic diseases compared with healthy participants. Patients with acute myocardial infarction and acute ischemic strokes were found to have higher PAI-1 levels [63]. In patients with metabolic syndrome, dietary restriction results in the lowering of the PAI-1 level [61].

During response to injury, plasmin and PAI-1 are involved in the wound healing process. Plasmin activates many latent growth factors and proteases, including metalloproteinases (MMP). The latter are responsible for the breakdown of the intercellular matrix. The failure of this process results in delayed healing and chronic inflammation with fibrosis [65]. PAI-1 enhances fibrosis in chronic inflammation in many organs [66,67,68]. In experimental animals, for example, transgenic mouse with the PAI-1 −/− genotype, fibrosis does not occur following injury, such as the bleomycin injury model, to the lung.

In COVID-19, both tPA and PAI-1 are involved in the complex pathway in which the spike protein of SARS-Co-2 attaches to a component of the renin–aldosterone–angiotensin system, ACE 2, during the invasion of the host cells [69,70,71]. First, plasmin and with other proteases, trypsin and transmembrane proteases (TMPRSS 2), facilitate the binding of the spike protein to ACE 2. Following binding, ACE is internalized and unable to process the breakdown of angiotensin II, leading to its excess. The excess of angiotensin II leads to an increase in PAI-1 [72]. This contributes to the hypercoagulable state seen in COVID-19. In addition, the excess of angiotensin II binds to its receptor angiotensin II receptor 1a, causing lung injury and leading to pulmonary edema with the formation of a hyaline membrane with fibrin in the alveoli [73,74]. Here, again, plasmin is involved in clearing fibrin. Furthermore, the diffuse alveolar damage with damaged type II alveolar cells leads to decreased surfactant, which results in the induction of the p53 pathway and increased PAI-1 [75].

In summary, the role of the fibrinolytic system is not limited to the resolution of fibrin and thrombi, but is involved a wide range of physiologic conditions and pathologic disorders. The intensive research carried out in recent years has revealed many new findings. This knowledge offers an opportunity for therapeutic development, particularly in the mitigation of the adverse effects of PAI-1. Greater understanding of these functions is essential for the management of many disorders.

## Figures and Tables

**Figure 1:**
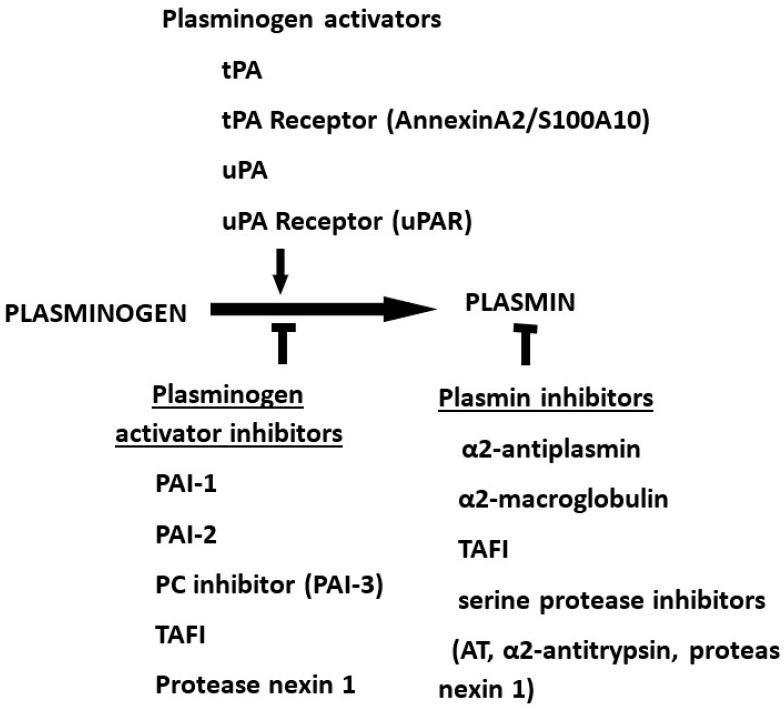
The fibrinolytic (plasminogen–plasmin) system.

**Table 1:** Physiological functions of the fibrinolytic system.

Embryogenesis Ovulation, menstruation
Pregnancy
Neuron growth
Brain function
Regulation of blood–brain barrier
Immunity
Wound healing
Senescence
Fibrosis

**Table 2:** Role of the fibrinolytic system in multiple disorders.

Neurologic disorders
Stroke/Hemorrhagic transformation
Degenerative disorders
Cancer proliferation, invasion/metastasis, angiogenesis
Vascular diseases
Atherosclerosis, myocardial infarction
Metabolic syndrome
Trauma
Fibrosis

## References

[1] Astrup T. (1968). Blood coagulation and fibrinolysis in tissue culture and tissue repair. Biochem. Pharmacol..

[2] Sherry S. (1964). Fibrinolysis and Clinical Medicine. Triangle.

[3] Sherry S. (1967). Fibrinolysis in health and disease. Med. Times.

[4] Sherry S. (1968). Fibrinolysis. Annu. Rev. Med..

[5] Kwaan H.C. (1972). Disorders of fibrinolysis. Med. Clin. N. Am..

[6] Kwaan H.C. (1979). Fibrinolysis–A perspective. Prog. Cardiovasc. Dis..

[7] Kwaan H.C. (1984). The role of fibrinolysis in disease processes. Semin. Thromb. Hemost..

[8] Miles L.A., Parmer R.J. (2013). Plasminogen receptors: The first quarter century. Semin. Thromb. Hemost..

[9] Cesarman G.M., Guevara C.A., Hajjar K.A. (1994). An endothelial cell receptor for plasminogen/tissue plasminogen activator (t-PA). II. Annexin II-mediated enhancement of t-PA-dependent plasminogen activation. J. Biol. Chem..

[10] Hajjar K.A., Jacovina A.T., Chacko J. (1994). An endothelial cell receptor for plasminogen/tissue plasminogen activator. I. Identity with annexin II. J. Biol. Chem..

[11] Hajjar K.A., Menell J.S. (1997). Annexin II: A novel mediator of cell surface plasmin generation. Ann. N. Y. Acad. Sci..

[12] O’Connell P.A., Madureira P.A., Berman J.N., Liwski R.S., Waisman D.M. (2011). Regulation of S100A10 by the PML-RAR-alpha oncoprotein. Blood.

[13] Ploug M., Ronne E., Behrendt N., Jensen A.L., Blasi F., Dano K. (1991). Cellular receptor for urokinase plasminogen activator. Carboxyl-terminal processing and membrane anchoring by glycosyl-phosphatidylinositol. J. Biol. Chem..

[14] Ny T., Liu Y.X., Ohlsson M., Jones P.B., Hsueh A.J. (1987). Regulation of tissue-type plasminogen activator activity and messenger RNA levels by gonadotropin-releasing hormone in cultured rat granulosa cells and cumulus-oocyte complexes. J. Biol. Chem..

[15] Seeds N.W., Williams B.L., Bickford P.C. (1995). Tissue plasminogen activator induction in Purkinje neurons after cerebellar motor learning. Science.

[16] Medcalf R.L. (2017). Fibrinolysis: From blood to the brain. J. Thromb. Haemost..

[17] Niego B., Lee N., Larsson P., De Silva T.M., Au A.E., McCutcheon F., Medcalf R.L. (2017). Selective inhibition of brain endothelial Rho-kinase-2 provides optimal protection of an in vitro blood-brain barrier from tissue-type plasminogen activator and plasmin. PLoS ONE.

[18] Yepes M., Sandkvist M., Moore E.G., Bugge T.H., Strickland D.K., Lawrence D.A. (2003). Tissue-type plasminogen activator induces opening of the blood-brain barrier via the LDL receptor-related protein. J. Clin. Investig..

[19] Fredriksson L., Lawrence D.A., Medcalf R.L. (2017). tPA Modulation of the Blood-Brain Barrier: A Unifying Explanation for the Pleiotropic Effects of tPA in the CNS. Semin. Thromb. Hemost..

[20] Parcq J., Bertrand T., Montagne A., Baron A.F., Macrez R., Billard J.M., Briens A., Hommet Y., Wu J., Yepes M. (2012). Unveiling an exceptional zymogen: The single-chain form of tPA is a selective activator of NMDA receptor-dependent signaling and neurotoxicity. Cell Death Differ..

[21] Hastings S., Myles P.S., Medcalf R.L. (2021). Plasmin, Immunity, and Surgical Site Infection. J. Clin. Med..

[22] Keragala C.B., Draxler D.F., McQuilten Z.K., Medcalf R.L. (2018). Haemostasis and innate immunity-a complementary relationship: A review of the intricate relationship between coagulation and complement pathways. Br. J. Haematol..

[23] Kolev K., Medcalf R.L. (2020). Editorial: Fibrinolysis in Immunity. Front. Immunol..

[24] Eren M., Boe A.E., Klyachko E.A., Vaughan D.E. (2014). Role of plasminogen activator inhibitor-1 in senescence and aging. Semin. Thromb. Hemost..

[25] Aillaud M.F., Pignol F., Alessi M.C., Harle J.R., Escande M., Mongin M., Juhan-Vague I. (1986). Increase in plasma concentration of plasminogen activator inhibitor, fibrinogen, von Willebrand factor, factor VIII:C and in erythrocyte sedimentation rate with age. Thromb. Haemost..

[26] Romer J., Bugge T.H., Pyke C., Lund L.R., Flick M.J., Degen J.L., Dano K. (1996). Plasminogen and wound healing. Nat. Med..

[27] Creemers E., Cleutjens J., Smits J., Heymans S., Moons L., Collen D., Daemen M., Carmeliet P. (2000). Disruption of the plasminogen gene in mice abolishes wound healing after myocardial infarction. Am. J. Pathol..

[28] Duffy M.J., Duggan C. (1997). Re: Urokinase and urokinase receptor: Association with in vitro invasiveness of human bladder cancer cell lines. J. Natl. Cancer Inst..

[29] Duffy M.J., Duggan C., Maguire T., Mulcahy K., Elvin P., McDermott E., Fennelly J.J., O’Higgins N. (1996). Urokinase plasminogen activator as a predictor of aggressive disease in breast cancer. Enzym. Protein.

[30] Duffy M.J., Duggan C., Mulcahy H.E., McDermott E.W., O’Higgins N.J. (1998). Urokinase plasminogen activator: A prognostic marker in breast cancer including patients with axillary node-negative disease. Clin. Chem..

[31] Duffy M.J., O’Grady P., Devaney D., O’Siorain L., Fennelly J.J., Lijnen H.J. (1988). Urokinase-plasminogen activator, a marker for aggressive breast carcinomas. Preliminary report. Cancer.

[32] Duffy M.J., Reilly D., O’Sullivan C., O’Higgins N., Fennelly J.J., Andreasen P. (1990). Urokinase-plasminogen activator, a new and independent prognostic marker in breast cancer. Cancer Res..

[33] Skelly M.M., Troy A., Duffy M.J., Mulcahy H.E., Duggan C., Connell T.G., O’Donoghue D.P., Sheahan K. (1997). Urokinase-type plasminogen activator in colorectal cancer: Relationship with clinicopathological features and patient outcome. Clin. Cancer Res..

[34] Look M., van Putten W., Duffy M., Harbeck N., Christensen I.J., Thomssen C., Kates R., Spyratos F., Ferno M., Eppenberger-Castori S. (2003). Pooled analysis of prognostic impact of uPA and PAI-1 in breast cancer patients. Thromb. Haemost..

[35] Harbeck N., Alt U., Berger U., Kruger A., Thomssen C., Janicke F., Hofler H., Kates R.E., Schmitt M. (2001). Prognostic impact of proteolytic factors (urokinase-type plasminogen activator, plasminogen activator inhibitor 1, and cathepsins B, D, and L) in primary breast cancer reflects effects of adjuvant systemic therapy. Clin. Cancer Res..

[36] Janicke F., Prechtl A., Thomssen C., Harbeck N., Meisner C., Untch M., Sweep C.G., Selbmann H.K., Graeff H., Schmitt M. (2001). Randomized adjuvant chemotherapy trial in high-risk, lymph node-negative breast cancer patients identified by urokinase-type plasminogen activator and plasminogen activator inhibitor type 1. J. Natl. Cancer Inst..

[37] Cantero D., Friess H., Deflorin J., Zimmermann A., Brundler M.A., Riesle E., Korc M., Buchler M.W. (1997). Enhanced expression of urokinase plasminogen activator and its receptor in pancreatic carcinoma. Br. J. Cancer.

[38] Gutova M., Najbauer J., Gevorgyan A., Metz M.Z., Weng Y., Shih C.C., Aboody K.S. (2007). Identification of uPAR-positive chemoresistant cells in small cell lung cancer. PLoS ONE.

[39] Soff G.A., Sanderowitz J., Gately S., Verrusio E., Weiss I., Brem S., Kwaan H.C. (1995). Expression of plasminogen activator inhibitor type 1 by human prostate carcinoma cells inhibits primary tumor growth, tumor-associated angiogenesis, and metastasis to lung and liver in an athymic mouse model. J. Clin. Investig..

[40] Kwaan H.C. (2020). The Central Role of Fibrinolytic Response in Trauma-Induced Coagulopathy: A Hematologist’s Perspective. Semin. Thromb. Hemost..

[41] Moore H.B., Cohen M.J., Moore E.E. (2020). Comment on “The S100A10 Pathway Mediates an Occult Hyperfibrinolytic Subtype in Trauma Patients”. Ann. Surg..

[42] Moore H.B., Moore E.E. (2019). TEG Lysis Shutdown Represents Coagulopathy in Bleeding Trauma Patients: Analysis of the PROPPR Cohort. Shock.

[43] Moore H.B., Moore E.E., Neal M.D., Sheppard F.R., Kornblith L.Z., Draxler D.F., Walsh M., Medcalf R.L., Cohen M.J., Cotton B.A. (2019). Fibrinolysis Shutdown in Trauma: Historical Review and Clinical Implications. Anesth. Analg..

[44] Moore H.B., Neeves K.B. (2019). Tranexamic acid for trauma: Repackaged and redelivered. J. Thromb. Haemost..

[45] Moore H.B., Walsh M., Kwaan H.C., Medcalf R.L. (2020). The Complexity of Trauma-Induced Coagulopathy. Semin. Thromb. Hemost..

[46] Walsh M., Fries D., Moore E., Moore H., Thomas S., Kwaan H.C., Marsee M.K., Grisoli A., McCauley R., Vande Lune S. (2020). Whole Blood for Civilian Urban Trauma Resuscitation: Historical, Present, and Future Considerations. Semin. Thromb. Hemost..

[47] Meizoso J.P., Karcutskie C.A., Ray J.J., Namias N., Schulman C.I., Proctor K.G. (2017). Persistent Fibrinolysis Shutdown Is Associated with Increased Mortality in Severely Injured Trauma Patients. J. Am. Coll. Surg..

[48] Roberts D.J., Kalkwarf K.J., Moore H.B., Cohen M.J., Fox E.E., Wade C.E., Cotton B.A. (2019). Time course and outcomes associated with transient versus persistent fibrinolytic phenotypes after injury: A nested, prospective, multicenter cohort study. J. Trauma Acute Care Surg..

[49] Sandrini L., Ieraci A., Amadio P., Zara M., Barbieri S.S. (2020). Impact of Acute and Chronic Stress on Thrombosis in Healthy Individuals and Cardiovascular Disease Patients. Int. J. Mol. Sci..

[50] Hoirisch-Clapauch S. (2022). Mechanisms affecting brain remodeling in depression: Do all roads lead to impaired fibrinolysis?. Mol. Psychiatry.

[51] Malemud C.J. (2006). Matrix metalloproteinases (MMPs) in health and disease: An overview. Front. Biosci..

[52] Strickland S., Beers W.H. (1976). Studies on the role of plasminogen activator in ovulation. In vitro response of granulosa cells to gonadotropins, cyclic nucleotides, and prostaglandins. J. Biol. Chem..

[53] Ohlsson R., Pfeifer-Ohlsson S. (1991). [Hereditary memory: Genomic imprinting and its importance for embryonal development and carcinogenesis]. Lakartidningen.

[54] Seeds N.W., Basham M.E., Haffke S.P. (1999). Neuronal migration is retarded in mice lacking the tissue plasminogen activator gene. Proc. Natl. Acad. Sci. USA.

[55] Melchor J.P., Strickland S. (2005). Tissue plasminogen activator in central nervous system physiology and pathology. Thromb. Haemost..

[56] Bennur S., Shankaranarayana Rao B.S., Pawlak R., Strickland S., McEwen B.S., Chattarji S. (2007). Stress-induced spine loss in the medial amygdala is mediated by tissue-plasminogen activator. Neuroscience.

[57] Su E.J., Fredriksson L., Geyer M., Folestad E., Cale J., Andrae J., Gao Y., Pietras K., Mann K., Yepes M. (2008). Activation of PDGF-CC by tissue plasminogen activator impairs blood-brain barrier integrity during ischemic stroke. Nat. Med..

[58] Boyd B.J., Galle A., Daglas M., Rosenfeld J.V., Medcalf R. (2015). Traumatic brain injury opens blood-brain barrier to stealth liposomes via an enhanced permeability and retention (EPR)-like effect. J. Drug Target..

[59] Lees K.R., Emberson J., Blackwell L., Bluhmki E., Davis S.M., Donnan G.A., Grotta J.C., Kaste M., von Kummer R., Lansberg M.G. (2016). Effects of Alteplase for Acute Stroke on the Distribution of Functional Outcomes: A Pooled Analysis of 9 Trials. Stroke.

[60] Whiteley W.N., Emberson J., Lees K.R., Blackwell L., Albers G., Bluhmki E., Brott T., Cohen G., Davis S., Donnan G. (2016). Risk of intracerebral haemorrhage with alteplase after acute ischaemic stroke: A secondary analysis of an individual patient data meta-analysis. Lancet Neurol..

[61] Kris-Etherton P.M., Stewart P.W., Ginsberg H.N., Tracy R.P., Lefevre M., Elmer P.J., Berglund L., Ershow A.G., Pearson T.A., Ramakrishnan R. (2020). The Type and Amount of Dietary Fat Affect Plasma Factor VIIc, Fibrinogen, and PAI-1 in Healthy Individuals and Individuals at High Cardiovascular Disease Risk: 2 Randomized Controlled Trials. J. Nutr..

[62] Sillen M., Declerck P.J. (2020). Targeting PAI-1 in Cardiovascular Disease: Structural Insights Into PAI-1 Functionality and Inhibition. Front. Cardiovasc. Med..

[63] Iacoviello L., Agnoli C., De Curtis A., di Castelnuovo A., Giurdanella M.C., Krogh V., Mattiello A., Matullo G., Sacerdote C., Tumino R. (2013). Type 1 plasminogen activator inhibitor as a common risk factor for cancer and ischaemic vascular disease: The EPICOR study. BMJ Open.

[64] Alessi M.C., Juhan-Vague I. (2006). PAI-1 and the metabolic syndrome: Links, causes, and consequences. Arter. Thromb. Vasc. Biol..

[65] Visse R., Nagase H. (2003). Matrix metalloproteinases and tissue inhibitors of metalloproteinases: Structure, function, and biochemistry. Circ. Res..

[66] Ghosh A.K., Quaggin S.E., Vaughan D.E. (2013). Molecular basis of organ fibrosis: Potential therapeutic approaches. Exp. Biol. Med..

[67] Ghosh A.K., Vaughan D.E. (2012). PAI-1 in tissue fibrosis. J. Cell. Physiol..

[68] Khan S.S., Shah S.J., Strande J.L., Baldridge A.S., Flevaris P., Puckelwartz M.J., McNally E.M., Rasmussen-Torvik L.J., Lee D.C., Carr J.C. (2021). Identification of Cardiac Fibrosis in Young Adults With a Homozygous Frameshift Variant in SERPINE1. JAMA Cardiol..

[69] Kwaan H.C., Lindholm P.F. (2021). The Central Role of Fibrinolytic Response in COVID-19-A Hematologist’s Perspective. Int. J. Mol. Sci..

[70] Kwaan H.C., Mazar A.P. (2022). More on the Source of D-Dimer in COVID-19. Thromb. Haemost..

[71] Walsh M.M., Khan R., Kwaan H.C., Neal M.D. (2021). Fibrinolysis Shutdown in COVID-19-Associated Coagulopathy: A Crosstalk among Immunity, Coagulation, and Specialists in Medicine and Surgery. J. Am. Coll. Surg..

[72] Vaughan D.E., Lazos S.A., Tong K. (1995). Angiotensin II regulates the expression of plasminogen activator inhibitor-1 in cultured endothelial cells. A potential link between the renin-angiotensin system and thrombosis. J. Clin. Investig..

[73] Kuba K., Imai Y., Rao S., Gao H., Guo F., Guan B., Huan Y., Yang P., Zhang Y., Deng W. (2005). A crucial role of angiotensin converting enzyme 2 (ACE2) in SARS coronavirus-induced lung injury. Nat. Med..

[74] Idell S., Kueppers F., Lippmann M., Rosen H., Niederman M., Fein A. (1987). Angiotensin converting enzyme in bronchoalveolar lavage in ARDS. Chest.

[75] Bhandary Y.P., Shetty S.K., Marudamuthu A.S., Ji H.L., Neuenschwander P.F., Boggaram V., Morris G.F., Fu J., Idell S., Shetty S. (2013). Regulation of lung injury and fibrosis by p53-mediated changes in urokinase and plasminogen activator inhibitor-1. Am. J. Pathol..

